# A multicountry, multicenter report to identify nutritional risks in female populations using the FIGO Nutrition Checklist

**DOI:** 10.1002/ijgo.70525

**Published:** 2025-09-09

**Authors:** Alex K. L. Taylor, Lucy Murphy, Sophie Callanan, Sarah Louise Killeen, Sharleen L. O'Reilly, Hema Divakar, Hamid Jan Bin Jan Mohamed, Mark A. Hanson, Fionnuala M. McAuliffe

**Affiliations:** ^1^ UCD Perinatal Research Center, School of Medicine, National Maternity Hospital University College Dublin Dublin Ireland; ^2^ Department of Clinical Nutrition and Dietetics National Maternity Hospital Dublin Ireland; ^3^ School of Agriculture and Food Science UCD Dublin 4 Ireland; ^4^ Obstetrics and Gynecology Divakars Specialty Hospital Bengaluru India; ^5^ Nutrition Program, School of Health Sciences Universiti Sains Malaysia Kubang Kerian Kelantan Malaysia; ^6^ Institute of Developmental Sciences, School of Human Development and Health, Faculty of Medicine University of Southampton Southampton UK

**Keywords:** FIGO Nutrition Checklist, non‐communicable diseases, nutrition, obesity, prevention, screening tool

## Abstract

**Objective:**

To identify potential nutritional risks for women using the FIGO Nutrition Checklist in relation to region, age and pregnancy/intention.

**Methods:**

A retrospective analysis was conducted using 1515 responses from the online version of the FIGO Nutrition Checklist available on the FIGO website. Participants who responded “No” to at least one dietary question were classified as at potential nutritional risk.

**Results:**

Most participants (1432/1515, 94.5%) answered “No” to at least one of the diet quality questions. Low fish intake was the most common potential nutritional risk (687/1515, 45.3%), followed by limited pulses consumption (577/1515, 38.1%). Over half of the participants aged 17–44 years (653/1265, 51.6%) reported not taking a folic acid supplement. More women of post‐reproductive age (>45 years) reported achieving the recommended standards for fruit and vegetables (166/236, 70.3% vs. 786/1265, 60.7%, *P* = 0.005), pulses (178/236, 75.4% vs. 749/1265, 59.2% vs. *P* < 0.001) and fish (147/236, 62.3% vs. 671/1265, 53% *P* = 0.009) compared with peak reproductive age participants. More non‐pregnant women (<45 years) reported meeting the recommended standards for wholegrain foods (391/480, 81.5% vs. 598/782, 76.5%, *P* = 0.037) and meat, poultry or eggs (416/480, 86.7% vs. 623/782, 79.7%, *P* = 0.002) compared with pregnant/planning pregnancy participants. When the five FIGO regions were compared, Africa and the Eastern Mediterranean were more likely to achieve all nutritional requirements followed by North America. Nearly half of the Asia Oceania region reported at least four potential nutritional risks (191/401, 47.6%), higher than other world regions.

**Conclusion:**

Nutritional risk is a global concern. The FIGO Nutrition Checklist helps healthcare professionals identify potential nutritional risks in women's diets and supports national and international implementation of nutrition guidelines.

## 1 INTRODUCTION

Suboptimal nutrition is a global issue with the WHO estimating that one in three people suffer from nutritional deficiency.^1^ The effect of maternal malnutrition can be seen through rising population weight gain resulting in a 43.8% prevalence of overweight and obesity globally.^2^, ^3^ The WHO reports an estimated 5 million non‐communicable disease (NCD) deaths are caused by higher‐than‐optimal body mass index (BMI, calculated as weight in kilograms divided by the square of height in meters).^2^ The importance of nutrition throughout the life course and its effect on NCD risk are widely discussed in the literature. The Developmental Origins of Health and Disease hypothesis explores how environmental influences, such as unbalanced nutrition, increase the risk of NCD and predispose offspring to a variety of metabolic disorders.^4^ Reproductive life stages are critical windows that drive maternal health and maternal malnutrition, both excessive and insufficient dietary intake are significant risk factors contributing to complications such as pre‐eclampsia, gestational diabetes mellitus and cardiovascular disease.^5^

Clinicians play a pivotal role in encouraging healthy lifestyle choices for women.^6^ Antenatal care is a unique opportunity to promote positive health change and reduce future NCD risk.^6^ Women report being interested in having the opportunity to discuss weight and nutrition,^7^ yet limited resources and time restraints are known barriers to engaging in such conversations. The International Federation of Obstetrics and Gynecology (FIGO) Nutrition Checklist is a validated and quick nutrition questionnaire used to collect simple, yet critical, nutrition information.^8^ The Checklist can also be used in the preconception period,^6^ as well as during pregnancy, to help shape future health trajectory for women and their offspring. It aids healthcare professionals in identifying potential nutritional risks and supports national and international nutrition in pregnancy guidelines.^8^ Clinicians can use the Checklist to facilitate diet and lifestyle conversations and support change through cost‐effective dietary interventions.^9^

While the Checklist is a validated tool, there have been no global reports on the nutritional data it can collect and whether the diets and health habits of different populations will align with country‐level data. Therefore, we aimed to identify potential nutritional risks of women who completed the Checklist across the five FIGO global regions and explore the additional impact of age and pregnancy intentions.

## 2 MATERIALS AND METHODS

### 2.1 Study design and population

This was a retrospective study that analyzed the FIGO Nutrition Checklist responses entered online (available at https://www.figo.org/figo‐resources/nutrition/figo‐nutrition‐checklist).

### 2.2 The FIGO Nutrition Checklist

The FIGO Nutrition Checklist was developed in 2015 by the FIGO Initiative on Adolescent, Preconception and Maternal Nutrition.^8^ It was designed as a simple 11‐item tool to identify unbalanced diets during the reproductive life stages that may require intervention. It can be completed by the woman alone or with a clinician, it is considered reliable when compared to a food frequency questionnaire and has been implemented in antenatal clinics in Ireland and Hong Kong.^8^, ^10^ The Checklist includes questions on self‐reported characteristics, such as country of origin, pregnancy status, weight and height, along with questions on dietary quality and markers of micronutrient status (folic acid supplementation, sun exposure [vitamin D], and iron levels). Diet quality and micronutrient status are assessed through “Yes/No” questions based on frequency of consumption/exposure. Participants who responded “No” to at least one dietary question were classified as at potential nutritional risk, warranting further evaluation.

### 2.3 Participant recruitment and selection

The Checklist was dispersed by healthcare professionals in clinical settings, such as antenatal clinics and at scientific meetings/conferences using a QR code, URL and social media. The online version is available in English, French and Spanish. Online responses were downloaded on August 22, 2024. A total of 2529 participants across five regions including 26 countries engaged with the Checklist during this period. Response rates varied by question and not all participants answered all questions.

### 2.4 Eligibility criteria and sample size

All participants engaging with the Checklist between July 15, 2022 and August 21, 2024 were downloaded. The inclusion criteria accepted only those participants with full data for the six diet quality questions, the micronutrient questions on folic acid supplementation and sun exposure. The final sample size was 1515 after these exclusions. Power and sample size calculations were not performed because the study is exploratory in design.

### 2.5 Ethical approval

While participants provided data on their health, diet and broad characteristics for age range, country of residence and pregnancy status, no identifying or personal data were collected. Women participated voluntarily on an opt‐in basis with consent obtained through the FIGO website terms and conditions agreement upon entry.

### 2.6 Statistical analysis

Statistical analyses were performed using IBM Statistical Package for Social Science version 29 for Windows. Normality was assessed for all continuous variables through histograms and normality tests. Normally distributed data are reported as mean and standard deviation (SD) and median and interquartile range (IQR) for non‐normal data. Categorical variables are reported as frequency and percentage (*n*, %). Descriptive statistics were generated for Checklist responses overall. Stratified analyses were performed to investigate the proportion of women at potential nutritional risk based on the following self‐reported characteristics: country of residence within the five FIGO Regions (Europe, Asia Oceania, North America, Latin America and Africa and Eastern Mediterranean); BMI according to the WHO criteria^11^; age (peak [17–44 years] versus post‐reproductive age [>45 years]); and pregnancy intention. Pearson Chi–squared test was carried out to determine all associations. A two‐sided *P* value less than 0.05 was considered statistically significant.

## 3 RESULTS

### 3.1 Demographics

Demographics are provided in Table 1. Most participants (906/1515, 59.8%) were between 25 and 39 years of age. The median BMI was 23.5, (21.3, 26.7 IQR) and 56.5% had BMI's in the healthy range. BMI categories were not associated with those who answered “No” to one or more of the diet quality questions (*P* = 0.016). Over half of the participants (54.1%, 812/1502) were pregnant or planning a pregnancy.

**TABLE 1:** Participant demographics.

*N* = 1515	Total (*N*, %)	Europe (*N*, %)	North America (*N*, %)	Latin America (*N*, %)	Asia Oceania (*N*, %)	Africa & Eastern Mediterranean (*N*, %)	Other (*N*, %)
Age (*N*, %)	*N* = 1495	*N* = 670 (44.8)	*N* = 89 (6)	*N* = 249 (16.7)	*N* = 400 (26.8)	*N* = 86 (5.8)	*N* = 1 (0.1)
17 and below	10 (0.7)	3 (0.4)	1 (1.1)	5 (2)	0 (0)	1 (1.2)	0 (0)
18–24	147 (9.8)	71 (10.6)	8 (9)	32 (12.9)	31 (7.8)	5 (5.8)	0 (0)
25–29	238 (15.9)	120 (17.9)	7 (7.9)	42 (16.9)	56 (14)	13 (15.1)	1 (100)
30–34	373 (24.9)	175 (26.1)	23 (25.8)	66 (26.5)	88 (22)	21 (24.4)	0 (0)
35–39	291 (19.5)	155 (23.1)	20 (22.5)	41 (16.5)	65 (16.3)	9 (10.5)	0 (0)
40–44	202 (13.5)	93 (13.9)	9 (10.1)	27 (10.8)	61 (15.3)	12 (14)	0 (0)
45–49	73 (4.9)	25 (3.7)	4 (4.5)	11 (4.4)	25 (6.3)	8 (9.3)	0 (0)
50+	161 (10.8)	28 (4.2)	17 (19.1)	25 (10)	74 (18.5)	17 (19.8)	0 (0)
BMI classification (*N*, %)	*N* = 1459	*N* = 653 (44.9)	*N* = 86 (5.9)	*N* = 246 (16.9)	*N* = 382 (26.3)	*N* = 86 (5.9)	*N* = 1 (0.1)
Underweight (<18.5)	84 (5.8)	45 (6.9)	6 (7.0)	6 (2.4)	22 (5.8)	5 (5.8)	0
Healthy weight (18.5–24.9)	825 (56.5)	410 (62.8)	39 (45.3)	137 (55.7)	197 (51.6)	38 (44.2)	1 (100)
Overweight (25–29.9)	371 (25.4)	127 (19.4)	28 (32.6)	74 (30.1)	115 (30.1)	26 (30.2)	0 (0)
Obesity Class I (30–34.9)	128 (8.8)	47 (7.2)	10 (11.6)	25 (10.2)	33 (8.6)	13 (15.1)	0 (0)
Obesity Class II (35–39.9)	30 (2.1)	15 (2.3)	2 (2.3)	2 (0.8)	8 (2.1)	3 (3.5)	0 (0)
Obesity Class III (>40)	21 (1.4)	9 (1.4)	1 (1.2)	2 (0.8)	7 (1.8)	1 (1.2)	0 (0)
Pregnancy status (*N*, %)	*N* = 1502	*N* = 673 (44.8)	*N* = 89 (5.9)	*N* = 253 (16.8)	*N* = 398 (26.5)	*N* = 88 (5.9)	*N* = 1 (0.1)
Pregnant/planning	812 (54.1)	375 (55.7)	47 (52.8)	150 (59.3)	198 (49.7)	41 (46.6)	1 (100)
Not pregnant/not planning	690 (45.9)	298 (44.3)	42 (47.2)	103 (40.7)	200 (50.3)	47 (53.4)	0 (0)
Breastfeeding status (*N*, %)	*N* = 697	*N* = 301 (43.2)	*N* = 42 (6.0)	*N* = 105 (15.1)	*N* = 201 (28.8)	*N* = 48 (6.9)	*N* = 0 (0)
Breastfeeding	50 (7.2)	27 (9)	2 (4.8)	4 (3.8)	11 (5.5)	6 (12.5)	0 (0)
Not breastfeeding	647 (92.8)	274 (91)	40 (95.2)	101 (96.2)	190 (94.5)	42 (87.5)	0 (0)

*Note*: BMI, calculated as weight in kilograms divided by the square of height in meters. All data are categorical and represented as *n* (%) based on total and FIGO region.

Abbreviation: BMI, body mass index, defined using the WHO classification.

### 3.2 Dietary quality

**FIGURE 1:**
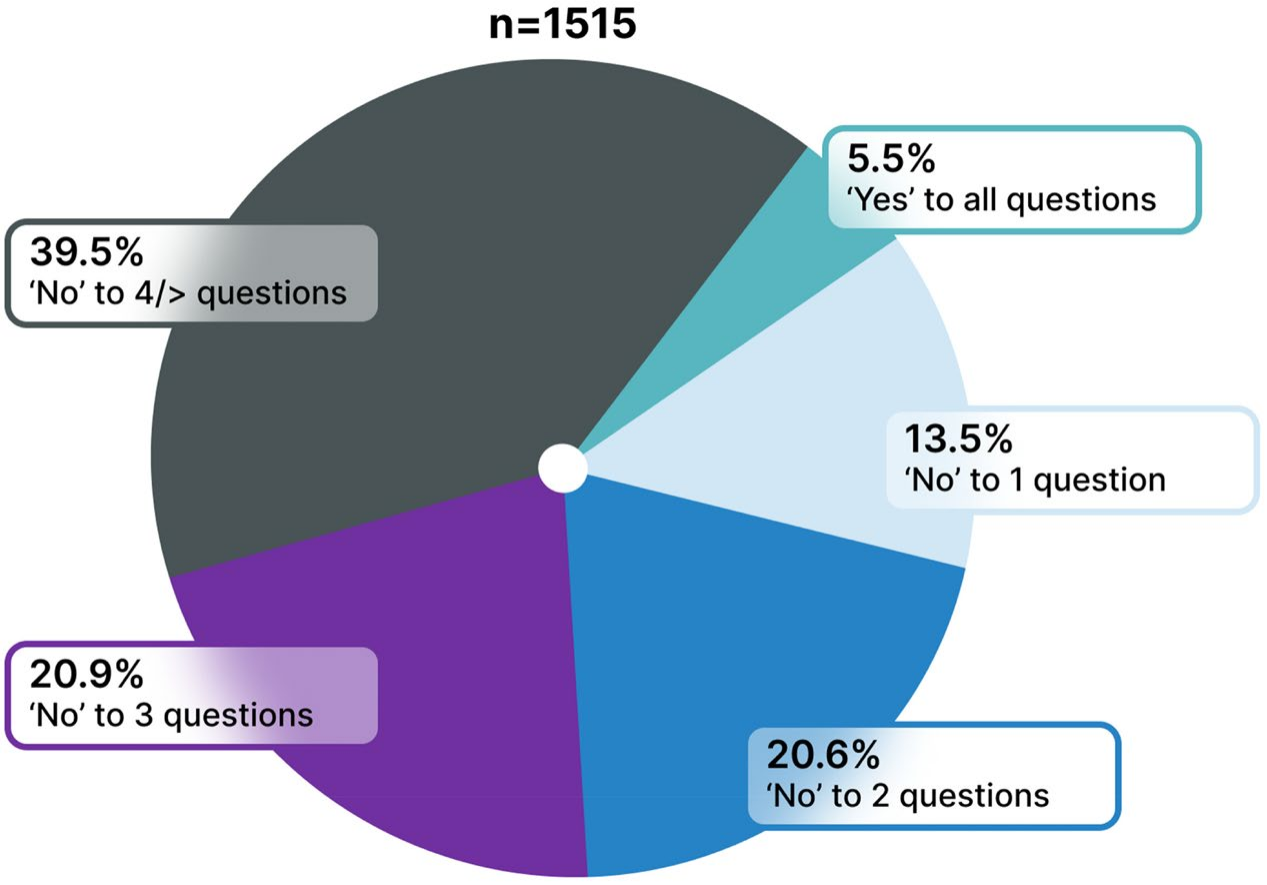
Participants' answers to all dietary questions in the FIGO Nutrition Checklist.

Most women (1432/1515, 94.5%) answered “No” to at least one nutrition question, suggesting potential nutritional risk as shown in Figure 1 and Table S1. The most common nutritional risks were low fish consumption (687/1515, 45.3%), limited pulses (577/1515, 38.1%) and fruit/vegetable intakes (574/1515, 37.9%). Consumption of low nutritional value foods was common with over one third reporting eating snacks, cakes, pastries, or sugar‐sweetened drinks more than five times a week (539/1515, 35.6%). Nearly half of all women reported not receiving the daily recommended sun exposure (729/1515, 48.1%). Anemia was diagnosed in one eighth of participants (139/840, 16.2%) with recent hemoglobin testing. Vegetarian (88/342, 25.7%) and flexitarian (82/342, 23.9%) dietary patterns were reported by over one fifth of participants (342/1515, 22.5%).

### 3.3 Peak versus post‐reproductive age groups

**FIGURE 2:**
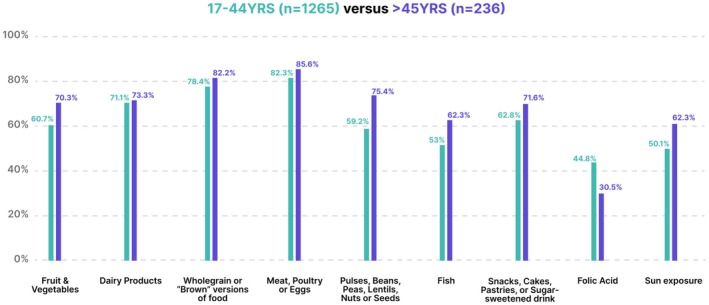
Peak reproductive (17–44 years) vs. post reproductive age (>45 years) achievement of dietary standards.

Peak reproductive age (17–44 years) were compared with those of post‐reproductive age women (>45 years) as shown in Figure 2 and Table S2. The post‐reproductive age group had significantly higher fruit/vegetables (166/236, 70.3% vs. 786/1265, 60.7%, *P* = 0.005), pulses (178/236, 75.4% vs. 749/1265, 59.2% vs. *P* < 0.001) and fish (147/236, 62.3% vs. 671/1265, 53% *P* = 0.009) consumption. Over half of the peak reproductive age group did not take folic acid supplements (653/1265, 51.6%).

### 3.4 Pregnant/planning to conceive versus non‐pregnant and not planning to conceive

**FIGURE 3:**
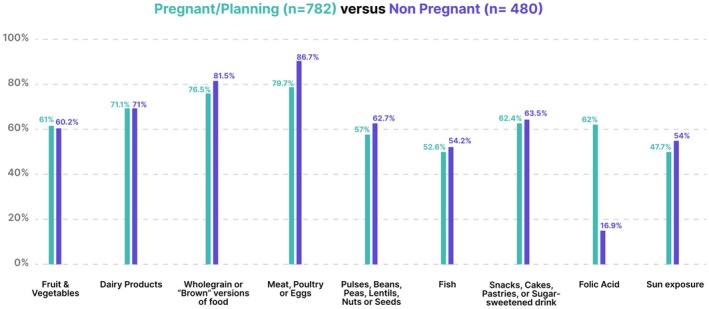
Pregnancy/planning pregnancy versus non‐pregnant (<45 years) achievement of dietary standards.

Participants under the age of 45 years who were pregnant or planning to conceive were compared with those not pregnant and not planning to conceive as shown in Figure 3 and Table S3. The non‐pregnant group had higher wholegrain (598/782, 76.5% vs. 391/480, 81.5%, *P* = 0.037) and meat, poultry or egg products (623/782, 79.7% vs. 417/480, 86.7%, *P* = 0.002) consumption. Folic acid supplementation was considerably higher in those planning pregnancy or pregnant (485/782, 62%) versus the non‐pregnant group (81/480, 16.9% *P* < 0.001).

### 3.5 Comparing the five FIGO regions

**FIGURE 4:**
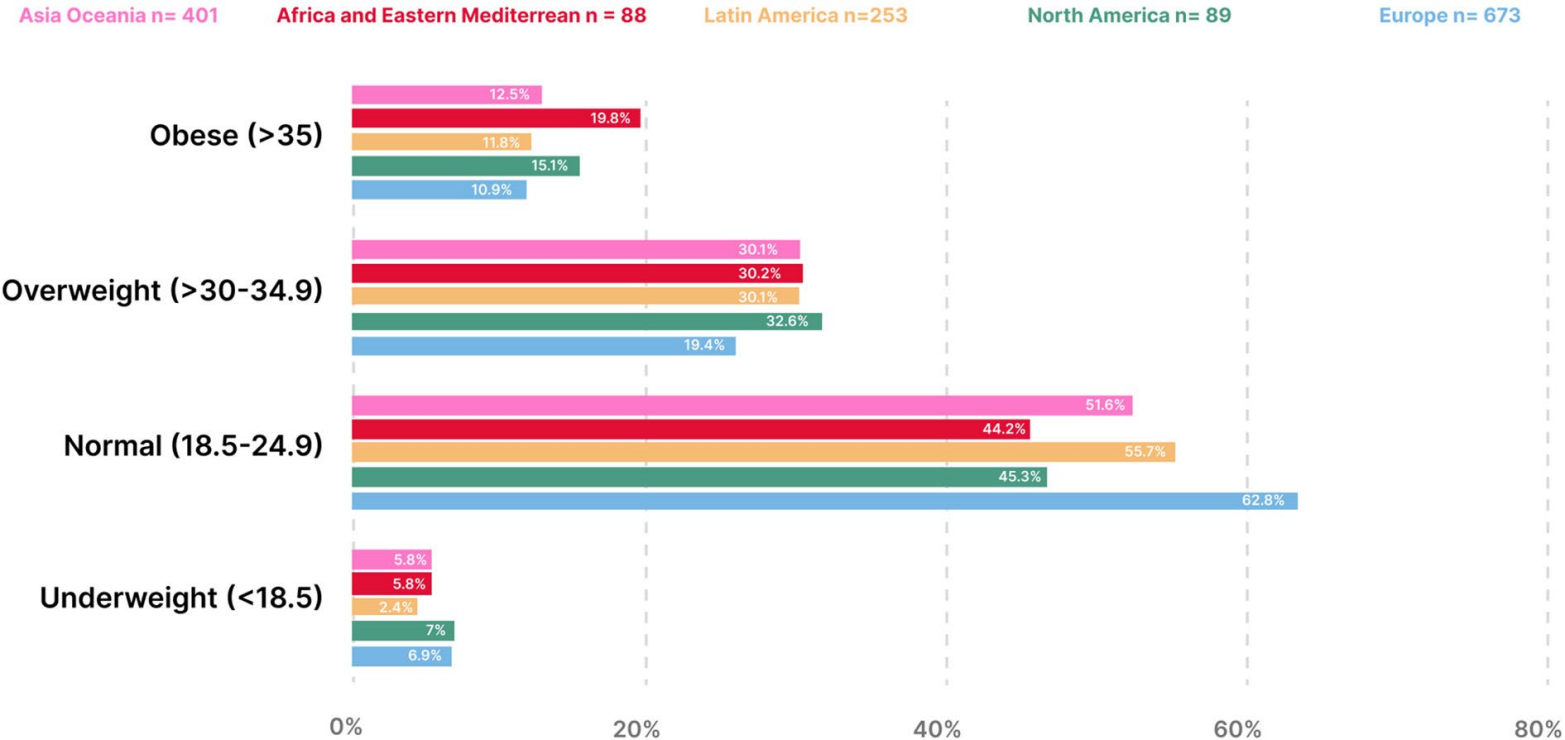
Body mass index (BMI, calculated as weight in kilograms divided by the square of height in meters) distribution across five FIGO regions.

**FIGURE 5:**
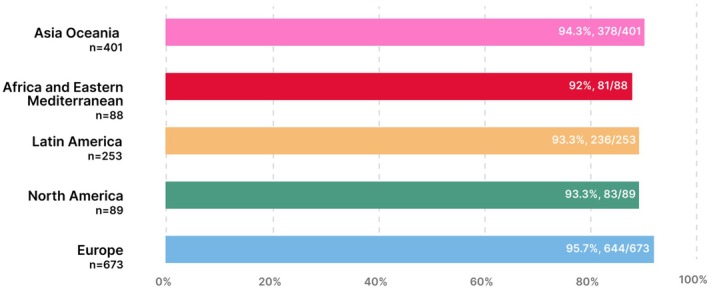
Five FIGO regions answered “No” to at least one nutritional risk question.

**FIGURE 6:**
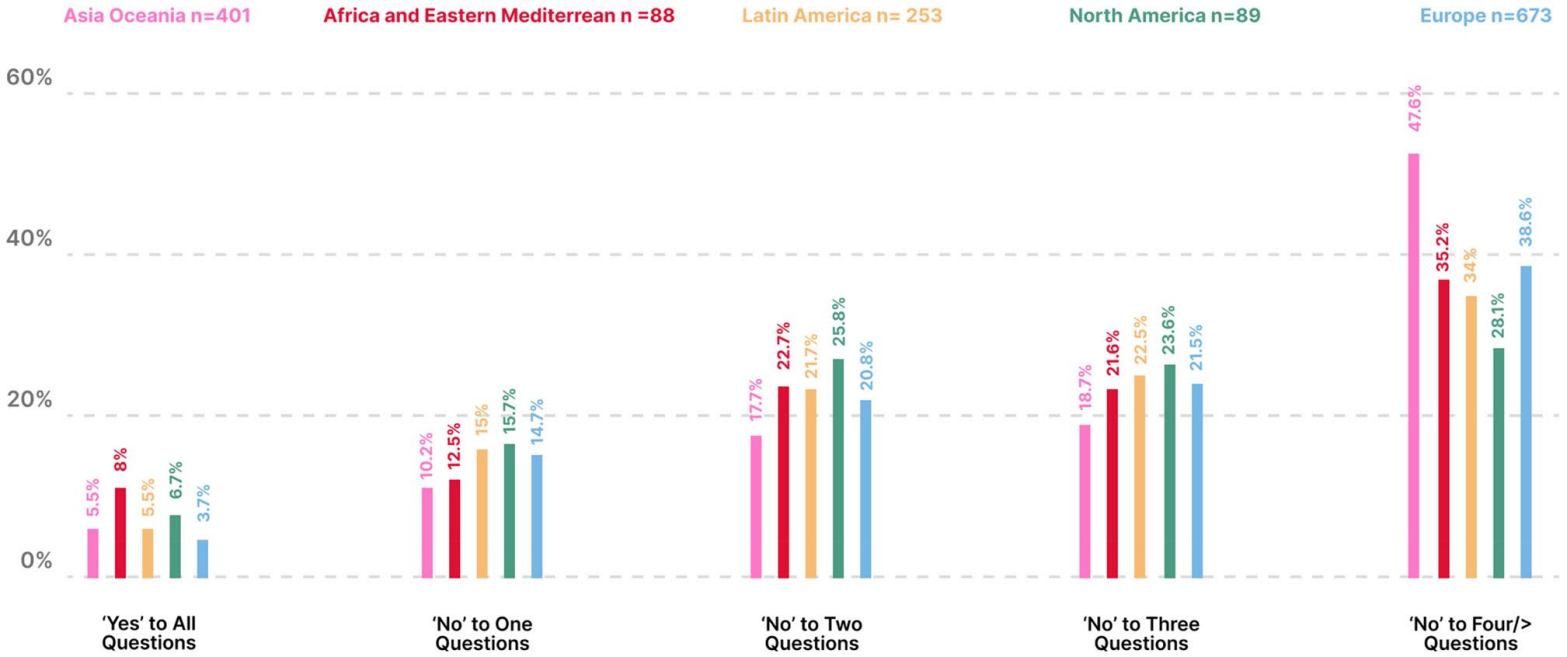
Comparing the five FIGO regions answers to all dietary questions in the FIGO Nutrition Checklist.

**FIGURE 7:**
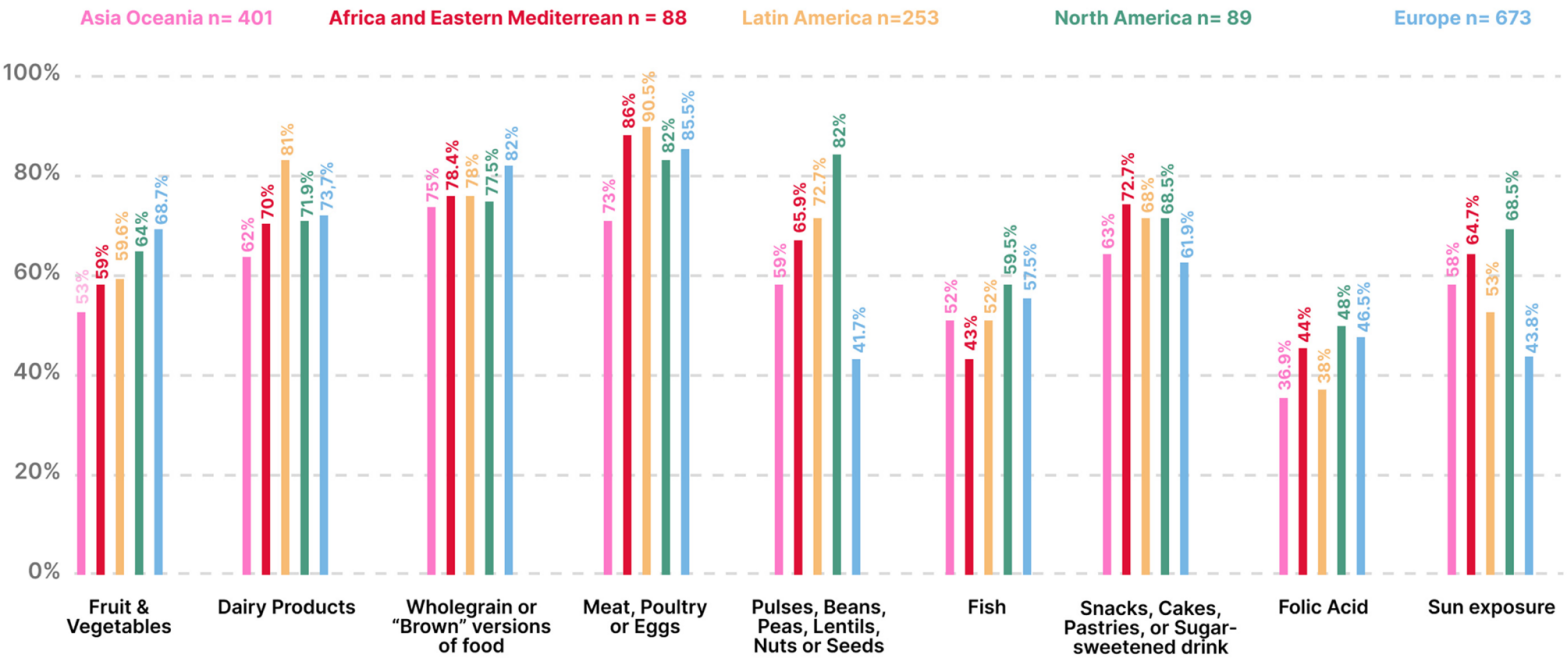
Comparing the five FIGO regions percentage of women reporting achievement of dietary standards.

**FIGURE 8:**
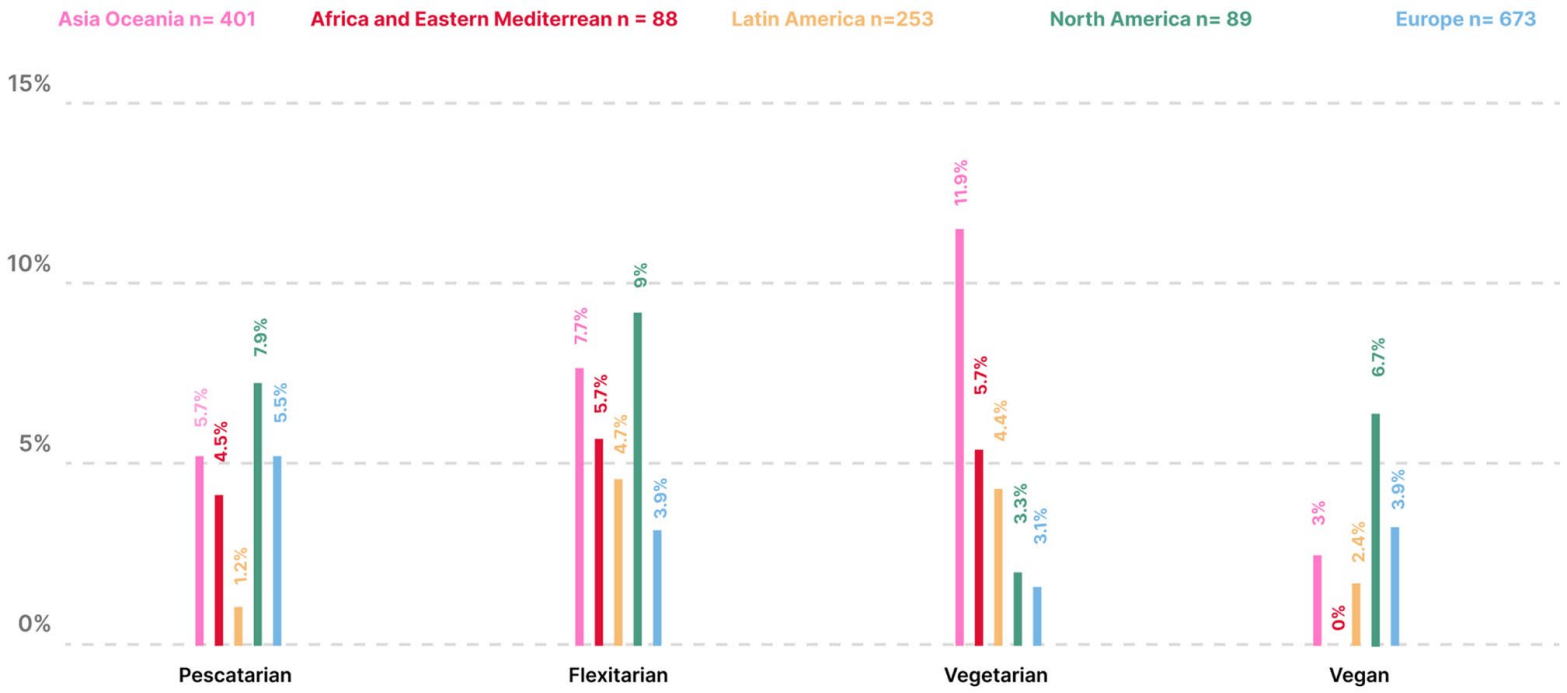
Percentage of special dietary restrictions across five FIGO regions.

Most participants were from Europe (673/1515, 44.7%) and Africa and Eastern Mediterranean had the least amount of participants (88/1515, 5.8%) (Table S4). The human development index characteristics of the countries of participants ranged from low (Mali, 0.419) to very high (Iceland, 0.972) as shown in Table S5. BMI was distributed evenly across all FIGO regions (Figure 4). Europe had the highest percentage of potential nutritional risk participants (95.7%) as shown in Figure 5 and Table S6. In all regions, most participants answered “No” to at least four questions with the Asia Oceania region at the highest risk (191/401, 47.6%) as shown in Figure 6 and Table S1. Over 40% of all regions reported low fish consumption, especially the Africa and Eastern Mediterranean region (50/88, 56.8%). Meat, poultry or eggs dietary standards were reported more commonly (73%–90.5%) (Table S7). The Latin American region reported a significantly higher consumption of dairy products compared to the Asia Oceania region (205/253, 81% vs. 249/401, 62%, *P* < 0.001). In the North American region, there was a significantly higher consumption of pulses compared to the European region (73/89, 82% vs. 281/673, 41.7%, *P* < 0.0001) (Figure 7). Asia Oceania reported the highest percentage of participants following a vegetarian diet (48/401, 11.9%) as shown in Figure 8 and Table S8.

## 4 DISCUSSION

The FIGO Nutritional Checklist is a useful tool to identify potential nutritional risk and encourage positive dietary change. The data reflects that nutrition is a global concern with a high percentage of potential nutritional risk worldwide. Better adherence to dietary standards were seen in the post‐reproductive age and the non‐pregnant participants. Throughout the five FIGO regions potential nutritional risk remained high. This study addresses global comparative data on the effectiveness of the Checklist which is limited in current literature.

### 4.1 Peak reproductive versus post reproductive age groups

Nutritional status during a woman's reproductive life is a significant contributor to maternal morbidity and a leading cause of childhood mortality and morbidity worldwide.^12^

Studies have reported that maternal nutrition at conception influences gamete function, placental development and long term maternal and offspring health.^13^ The impact of maternal nutrition on fetal epigenetic mechanisms affecting gene expression and thus developmental plasticity links maternal periconception nutrition to an offspring's long‐term health trajectory, including metabolic disease, immune function and neurodevelopment.^13^ Folic acid supplementation's role in the reduction and prevention of neural tube defects is widely acknowledged and folate levels are linked to one‐carbon metabolism and hence DNA methylation which underpins epigenetic processes.^13^, ^14^ Over half of peak reproductive age participants in this study reported not taking folic acid supplementation, which is concerning given an estimated 208 million pregnancies occur annually, with 46% of those being unplanned.^13^ We also identified poorer dietary patterns in this group, which highlights that further work is required to improve diet and nutrition pre‐conception if we want to improve long‐term health in both women and their children.^15^

### 4.2 Pregnant or planning to conceive versus non‐pregnant and not planning to conceive

Nutritional status is one of the few modifiable risk factors affecting fertility.^16^ Suboptimal nutrition impacts ovulatory function, embryo quality and likelihood of implantation.^17^ Body habitus and other nutritional factors are linked to nearly half of all ovulation disorders, the leading cause of female infertility.^16^ A systematic review assessing preconception diet and fertility reports the Mediterranean diet as the most consistent association with improved pregnancy rates.^18^ We found lower adherence to this diet in the pregnant/planning to conceive group compared to those not planning pregnancy, especially in wholegrain and pulses consumption. Thus identifying a modifiable risk factor for potential fertility challenges. Reduction of trans‐fatty acids, saturated fatty acids, and consumption of low nutritional value foods are associated with higher pregnancy rates, irrespective of other factors such as age, BMI and lifestyle.^18^ Major characteristics of the Western diet include high intakes of added sugars, saturated and trans‐fats.^17^ This is reflected in our data with over one third of our participants consuming snacks, cakes, pastries or sugar sweetened beverages more than five times a week. Both groups had high percentage of those consuming 2–3 servings of red meat per week.

### 4.3 Fish

Fish provide key nutrients for a healthy pregnancy, specifically fetal neurocognitive development.^19^ Iodine, long chain omega‐3 polyunsaturated fatty acids and vitamins A, D and B12, are all found in fish and important for fetal development.^20^ Nearly one quarter of our participants reported special dietary requirements. The suboptimal fish intake may be due this as well as cultural, ethical, accessibility or affordability barriers. However, the pulses category is the second most common potential nutritional risk. We, therefore, cannot be reassured that those not eating fish are consuming sources of omega 3 plant‐based alternatives commonly found in flaxseed, chia seeds, hemp seeds and walnuts.^21^ Alpha‐linolenic acid (ALA) is the simplest omega‐3 fatty acid and is found in plant sources, and must be converted to the active form eicosapentaenoic acid (EPA) and docosahexaenoic acid (DHA), found in fish. EPA and DHA can be used by the body immediately and are more beneficial than ALA, which need to be consumed in large amounts for similar benefits.^22^ Discussion about the health benefits of fish would help promote nutritional adequacy and quality.

### 4.4 Comparing the five FIGO regions

Dietary patterns naturally vary across different populations, with contributing factors such as age, socioeconomic status, culture as well as the affordability and availability of different foods.^23^ Even within each FIGO region dietary profiles will vary and our data reflects the diversity of food patterns across the globe.^23^ The FIGO Nutrition Checklist appropriately highlights potential nutritional risk across the five FIGO regions. Studies focusing on the Indian population conclude that most diets are vegetarian^24^ and report Asia Oceania as the highest percentage of participants following a vegetarian diet. Most protein consumption within the Indian population is derived from pulses^24^ and our data reflects this with nearly two thirds of the Asia Oceania region achieving dietary standards in the pulses category. In the African and Eastern Mediterranean region, flat‐bread, couscous and rice are staples in the diet and fish is not frequently consumed yet present in dietary guidelines for this region.^25^ This is also represented in our data as fish intake is the highest potential nutritional risk in the Africa and Eastern Mediterranean region. Economy, climate, politics and religion also impact dietary factors throughout cultures and can pose a barrier to the accessibility and affordability of food in different regions.^25^

### 4.5 Clinical implications

There is a need for more routine nutrition assessments to help prevent obesity and NCDs such as diabetes and cardiovascular disease. The Checklist can be incorporated in routine care including preconception counseling and antenatal appointments. The Checklist aids healthcare professionals in identifying potential nutritional risks and should be widely adopted to support national and international guidelines, to ensure health benefits for women and children.

### 4.6 Strengths and limitations

This study had a large and globally diverse sample that captures potential nutritional risks across five regions including 26 countries. The age range and cultural diversity allow for a unique global view of nutrition. The limitations include possible sampling bias. Response rates varied across world regions, for example, Europe had 673 responses while Africa and Eastern Mediterranean had 88. Only women with access to the online version of the Checklist were included, which was mainly shared at academic meetings and in clinical settings and may underrepresent those living in rural areas and with a lower educational attainment. The results should be interpreted with caution because some results may be attributed to unmeasured or residual confounding factors such as income, which should be explored in future work. The Checklist does not consider the potential for participants obtaining dietary requirements in other solutions such as nutritional supplements or meal replacements. Another limitation is the qualitative nature of the Checklist questions as it does not measure actual quantitative food intakes. It is not possible for a single tool to accurately reflect the myriad of population‐specific dietary recommendations so the Checklist is only able to reflect the patterns present in dietary guidelines internationally. The Checklist sets a minimum standard of nutrition intake and acts as a platform to discuss a woman's nutritional needs.

### 4.7 Future research

Future studies could include modification of the FIGO Nutrition Checklist with additional subsections to facilitate those with specific dietary and cultural requirements and additional questions to allow further information to be given if they have answered “No” to dietary questions. Additional questions to determine economic status would be beneficial for analyzing purchasing power of each participant. Further education for healthcare professionals to implement the Checklist into routine care and further assess its use as an education tool in clinical settings.

## 5 CONCLUSION

Nutritional risk is an important issue for women of reproductive age. Our findings confirm that there is suboptimal nutrition in women worldwide, with 94.5% potentially at nutritional risk. The data identified potential nutritional risk in women at peak reproductive age and the pregnant/planning pregnancy participants of the population. Low fish intake is the greatest potential dietary risk across the FIGO regions. The FIGO Nutrition Checklist can identify nutritional risks and enable healthcare professionals to promote healthy lifestyle choices in women of reproductive age and beyond.

## AUTHOR CONTRIBUTIONS

FMcA and MH conceived the study. AKL Taylor took the lead in writing the manuscript, designing figures and data analysis. LM analyzed the data and contributed to writing and editing of manuscript. SC advised on statistical analysis and contributed to the manuscript preparation. SLK, SOR, HD, HJBJM contributed to shaping and editing manuscript. All authors approved the final version of the manuscript and agree to be accountable for all aspects of the work.

## FUNDING INFORMATION

The authors state that no funding was involved.

## CONFLICT OF INTEREST STATEMENT

The authors declare no conflict of interest.

## Supporting information


Table S1.
Table S2.
Table S3.
Table S4.
Table S5.
Table S6.
Table S7.
Table S8.

## Data Availability

The data that support the findings of this study are available from the corresponding author, F M McAuliffe, upone reasonable request.
