# Supplementary material for: A multicountry, multicenter report to identify nutritional risks in female populations using the FIGO Nutrition Checklist
Source: Int J Gynaecol Obstet. 2025 Sep 9;172(3):1610–8. doi: 10.1002/ijgo.70525 (PMC12936633; doi:10.1002/ijgo.70525)
Supplement: Supplementary file 1 — Table S1. Table S2. Table S3. Table S4. Table S5. Table S6. Table S7. Table S8. [file IJGO-172-1610-s001.docx]

| **Supplementary Table 1: Participants’ responses to the FIGO Nutrition Checklist according to the FIGO Five regions. N = 1505** | | | | | | | | |
| --- | --- | --- | --- | --- | --- | --- | --- | --- |
|  | **Total** | **Africa and Eastern Mediterranean** | **Asia Oceania** | **Europe** | **Latin America** | **North America** | **Other** | **P-value** |
|  | N = 1505 | N = 88 | N = 401 | N = 673 | N = 253 | N = 89 | N = 1 |  |
| ‘Yes’ to all questions, n(%) | 82 (5.4%) | 7(8%)_a_ | 22(5.5%)_a_ | 25 (3.7%)_a_ | 14 (5.5%)_a_ | 6 (6.7%)_a_ | 0_a_ |  |
| ‘No’ to 1 question, n(%) | 203 (13.5%) | 11 (12.5%)_a, b_ | 41(10.2%)_a_ | 99 (14.7%)_b_ | 38 (15%)_a, b_ | 14 (15.7%)_a, b_ | 0_a,b_ | <0.001 |
| ‘No’ to 2 questions, n(%) | 309 (13.5%) | 20 (22.7%)_a_ | 71(17.7%)_a_ | 140 (20.8%)_a_ | 55(21.7%)_a_ | 23 (25.8%) _a_ | 0 _a_ |  |
| ‘No’ to 3 questions, n(%) | 317 (21.1%) | 19 (21.6%) _a_ | 75(18.7%) _a_ | 145 (21.5%) _a_ | 57 (22.5%) _a_ | 21 (23.6%) _a_ | 0 _a_ |  |
| >3 ‘No’ responses, n (%) | 594 (39.5%) | 31 (35.2%) | 191 (47.6%) | 260 (38.6%) | 86 (34%) | 25 (28.1%) | 1 (100%) | <0.001 |
| ≤3 ‘No’ responses, n (%) | 911 (60.5%) | 57 (64.8%) | 210 (52.4%) | 413 (61.4%) | 167 (66%) | 64 (71.9%) | 0 (0%) |  |
| *Each subscript letter denotes a subset of FIGO regions whose column proportions do not differ significantly from each other at the 0.05 significance level* | | | | | | | | |

| **Supplementary Table 2: Dietary standards in those of peak reproductive and post reproductive age n=1501** | | | |
| --- | --- | --- | --- |
|  | **Peak reproductive age**  **17-44** | **Post reproductive age**  **45+** | **P Value** |
|  | N = 1265 | N = 236 |  |
| ***Do you eat three or more servings of fruit or vegetables (dried, juice, frozen, tinned or fresh) each day?*** | | | |
| ‘Yes’, n (%) | 768(60.7%) | 166(70.3%) | **0.005** |
| ‘No’, n(%) | 497(39.3%) | 70(29.7%) |  |
| ***Do you eat at least one serving of dairy products (milk/milky drinks, yoghurt, cheese) each day?*** | | | |
| ‘Yes’, n (%) | 900(71.1%) | 173(73.3%) | 0.5 |
| ‘No’, n(%) | 365(28.9%) | 63(26.7%) |  |
| ***Do you eat at least one serving of wholegrain or "brown" versions of foods such as bread, rice, pasta, crackers, cereals, oats, millet or wheat each day?*** | | | |
| ‘Yes’, n (%) | 992(78.4%) | 192(82.2%) | 0.190 |
| ‘No’, n(%) | 273(21.6%) | 42(17.8%) |  |
| ***Do you eat at least two or three servings of meat, poultry (chicken or turkey) or eggs each week?*** | | | |
| ‘Yes’, n (%) | 1041(82.3%) | 202(85.6%) | 0.217 |
| ‘No’ n(%) | 224(17.7%) | 34(14.4%) |  |
| ***Do you eat at least two or three servings of pulses, beans, peas, lentils, nuts or seeds each week?*** | | | |
| ‘Yes’, n (%) | 749(59.2%) | 178(75.4%) | **<0.001** |
| ‘No’ n(%) | 516(40.8%) | 58(24.6%) |  |
| ***Do you eat at least one serving of fish each week?*** | | | |
| ‘Yes’, n (%) | 671(53%) | 147(62.3%) | 0.009 |
| ‘No’ n(%) | 594(47%) | 89(37.7%) |  |
| ***Do you limit eating packaged snacks, cakes, pastries, or sugar-sweetened drinks to less than five times a week?*** | | | |
| ‘Yes’, n (%) | 795(62.8%) | 169(71.6%) | 0.010 |
| ‘No’ n(%) | 470(37.2%) | 67(28.4%) |  |
| *Do you take folic acid?* | | | |
| ‘Yes’, n (%) | 567(44.8%) | 72(30.5%) | **<0.001** |
| ‘No’ n(%) | 653(51.6%) | 154(65.3%) |  |
| ‘I don’t know’ n(%) | 45(3.6%) | 10(4.2%) |  |
| ***Do you get regular exposure to the sun on your face, arms and hands for at least 10-15 minutes a day?*** | | | |
| ‘Yes’, n (%) | 634(50.1%) | 147(62.3%) | **0.002** |
| ‘No’ n(%) | 547(43.2%) | 79(33.5%) |  |
| ‘I don’t know’ n(%) | 84(6.6%) | 10(4.2%) |  |
| *Data represented as categorical variables reported as n, (%) based on age group. Values determined using statistical significance p-value <0.05.* | | | |

| **Supplementary Table 3: Achieving dietary standards in those of peak reproductive age.** **N = 1262** | | | |
| --- | --- | --- | --- |
|  | **Peak reproductive age (<45 years old)** | | **P-value** |
|  | **Pregnant or planning a**  **pregnancy** | **Not pregnant or not planning a pregnancy** |  |
|  | N = 782 | N = 480 |  |
| ***Do you eat three or more servings of fruit or vegetables (dried, juice, frozen, tinned or fresh) each day?*** | | | |
| ‘Yes’, n (%) | 477 (61%) | 289 (60.2%) | 0.781 |
| ***Do you eat at least one serving of dairy products (milk/milky drinks, yoghurt, cheese) each day?*** | | | |
| ‘Yes’, n (%) | 556 (71.1%) | 341 (71%) | 0.982 |
| ***Do you eat at least one serving of wholegrain or "brown" versions of foods such as bread, rice, pasta, crackers, cereals, oats, millet or wheat each day?*** | | | |
| ‘Yes’, n (%) | 598 (76.5%) | 391 (81.5%) | 0.037 |
| ***Do you eat at least two or three servings of meat, poultry (chicken or turkey) or eggs each week?*** | | | |
| ‘Yes’, n (%) | 623 (79.7%) | 416 (86.7%) | 0.002 |
| ***Do you eat at least two or three servings of pulses, beans, peas, lentils, nuts or seeds each week?*** | | | |
| ‘Yes’, n (%) | 446 (57%) | 301 (62.7%) | 0.046 |
| ***Do you eat at least one serving of fish each week?*** | | | |
| ‘Yes’, n (%) | 411 (52.6%) | 260 (54.2%) | 0.578 |
| ***Do you limit eating packaged snacks, cakes, pastries, or sugar-sweetened drinks to less than five times a week?*** | | | |
| ‘Yes’, n (%) | 488 (62.4%) | 305 (63.5%) | 0.685 |
| ***Do you take folic acid?*** | | | |
| ‘Yes’, n (%) | 485 (62%) | 81 (16.9%) | <0.001 |
| ***Do you get regular exposure to the sun on your face, arms and hands for at least 10-15 minutes a day?*** | | | |
| ‘Yes’, n (%) | 373 (47.7%) | 259 (54%) | 0.091 |
| *Data represented as categorical variables reported as n, (%) based on those in the peak reproductive age group (<45 years) and pregnancy intention. Values determined using statistical significance p-value <0.05.* | | | |

| **Supplementary Table 4: Percentage of those who completed the FIGO Nutrition checklist from each country separated by FIGO Regions** | | |
| --- | --- | --- |
| **African & Eastern Mediterranean Region** | | |
| **Total participants** | **Countries included n=30** | **Answered all nutrition questions, n(%)** |
| **88(5.8%)** | United Arab Emirates | 4(0.3) |
|  | Saudi Arabia | 2(0.1) |
|  | Turkey | 4(0.3) |
|  | Israel | 7(0.5) |
|  | Qatar | 1(0.1) |
|  | Oman | 1(0.1) |
|  | Kuwait | 1(0.1) |
|  | Egypt | 2(0.1) |
|  | Lebanon | 1(0.1) |
|  | South Africa | 9(0.6) |
|  | Gabon | 1(0.1) |
|  | Botswana | 7(0.5) |
|  | Iraq | 5(0.3) |
|  | Namibia | 2(0.1) |
|  | The Democratic Republic of The Congo | 2(0.1) |
|  | Kenya | 4(0.3) |
|  | Ghana | 3(0.2) |
|  | Angola | 1(0.1) |
|  | Cameroon | 1(0.1) |
|  | Uganda | 1(0.1) |
|  | Rwanda | 1(0.1) |
|  | Nigeria | 2(0.1) |
|  | United Republic of Tanzania | 1(0.1) |
|  | Pakistan | 13(0.9) |
|  | Malawi | 1(0.1) |
|  | Ethiopia | 5(0.3) |
|  | Afghanistan | 5(0.3) |
|  | Yemen | 1(0.1) |
|  | Burkina Faso | 1(0.1) |
|  | Mali | 1(0.1) |
| **European Region** | | |
| **Total participants, n(%)** | **Countries included, n=42** | **Answered all nutrition questions, n(%)** |
| **672(44.3%)** | Iceland | 2(0.1) |
|  | Switzerland | 11(0.7) |
|  | Norway | 5(0.3) |
|  | Gibraltar | 1(0.1) |
|  | Sweden | 5(0.3) |
|  | Germany | 21(1.4) |
|  | Netherlands | 5(0.3) |
|  | Belgium | 9(0.6) |
|  | Ireland | 156(10.3) |
|  | Finland | 5(0.3) |
|  | United Kingdom | 74(4.9) |
|  | Slovenia | 9(0.6) |
|  | Austria | 2(0.1) |
|  | Malta | 2(0.1) |
|  | Luxembourg | 3(0.2) |
|  | Denmark | 1(0.1) |
|  | France | 64(4.2) |
|  | Spain | 34(2.2) |
|  | Czech Republic | 2(0.1) |
|  | Italy | 22(1.5) |
|  | Andorra | 1(0.1) |
|  | Cyprus | 1(0.1) |
|  | Greece | 7(0.5) |
|  | Poland | 8(0.5) |
|  | Estonia | 7(0.5) |
|  | Lithuania | 4(0.3) |
|  | Portugal | 6(0.4) |
|  | Croatia | 3(0.2) |
|  | Latvia | 2(0.1) |
|  | Slovakia | 3(0.2) |
|  | Romania | 4(0.3) |
|  | Bulgaria | 3(0.2) |
|  | Georgia | 2(0.1) |
|  | Serbia | 1(0.1) |
|  | Russia | 89(5.9) |
|  | Belarus | 12(0.8) |
|  | Macedonia | 1(0.1) |
|  | Armenia | 3(0.2) |
|  | Albania | 3(0.2) |
|  | Azerbaijan | 1(0.1) |
|  | Moldova | 7(0.5) |
|  | Ukraine | 72(4.8) |
| **Asia Oceania Region** | | |
| **Total participants, n(%)** | **Countries included, n=28** | **Answered all nutrition questions, n(%)** |
| **403(26.6%)** | Australia | 62(4.1) |
|  | Hong Kong | 11(0.7) |
|  | Singapore | 5(0.3) |
|  | New Zealand | 6(0.4) |
|  | Korea | 2(0.1) |
|  | Japan | 23(1.5) |
|  | Taiwan | 10(0.7) |
|  | Guam | 1(0.1) |
|  | Brunei Darussalam | 1(0.1) |
|  | Kazakhstan | 5(0.3) |
|  | Malaysia | 14(0.9) |
|  | Thailand | 1(0.1) |
|  | China | 15(1.0) |
|  | Sri Lanka | 2(0.1) |
|  | Vietnam | 6(0.4) |
|  | Mongolia | 1(0.1) |
|  | Uzbekistan | 2(0.1) |
|  | Fiji | 4(0.3) |
|  | Indonesia | 8(0.5) |
|  | Philippines | 93(6.1) |
|  | Kyrgyzstan | 2(0.1) |
|  | American Samoa | 4(0.3) |
|  | India | 112(7.4) |
|  | Bangladesh | 1(0.1) |
|  | Nepal | 7(0.5) |
|  | Myanmar | 1(0.1) |
|  | Papua New Guinea | 1(0.1) |
|  | United States Minor Outlying Islands | 1(0.1) |
| **North American Region** | | |
| **Total participants, n(%)** | **Countries included n=2** | **Answered all nutrition questions, n(%)** |
| **90(5.9%)** | Canada | 9(0.6) |
|  | United States | 80(5.3) |
| **Latin American Region** | | |
| **Total participants, n(%)** | **Countries included, n=23** | **Answered all nutrition questions, n(%)** |
| **254(16.7%)** | Aruba | 1(0.1) |
|  | Chile | 9(0.6) |
|  | Argentina | 14(0.9) |
|  | Uruguay | 6(0.4) |
|  | Antigua and Barbuda | 1(0.1) |
|  | Puerto Rico | 1(0.1) |
|  | Panama | 1(0.1) |
|  | Costa Rica | 2(0.1) |
|  | Peru | 6(0.4) |
|  | Mexico | 94(6.2) |
|  | Colombia | 23(1.5) |
|  | Brazil | 66(4.4) |
|  | Ecuador | 5(0.3) |
|  | Guyana | 2(0.1) |
|  | Dominican Republic | 3(0.2) |
|  | Cuba | 2(0.1) |
|  | Paraguay | 3(0.2) |
|  | Bolivia | 1(0.1) |
|  | Belize | 1(0.1) |
|  | Venezuela | 7(0.5) |
|  | Guatemala | 3(0.2) |
|  | Monserrat | 1(0.1) |
|  | Guadeloupe | 1(0.1) |
| **Other countries included that did not fall into the FIGO Five Regions** | | |
| **Total participants, n(%)** | **Countries included, n=1** | **Answered all nutrition questions, n(%)** |
| **1(0.1%)** | Antarctica | 1(0.1) |

| **Supplementary Table 5 : Showing the Human Development Index characteristics of the countries of participants who completed the FIGO Nutrition Checklist** | | | | | |
| --- | --- | --- | --- | --- | --- |
| **Region** | **Country** | **HDI 2023** | **Current HDI Tier** | **GDP per capita, purchasing power parity (thousand, $)** | **Year** |
| **Africa & Eastern Mediterranean** | United Arab Emirates | 0.940 | Very High | 77,958.9 | 2024 |
|  | Saudi Arabia | 0.900 | Very High | 71,243.4 | 2024 |
|  | Turkey | 0.853 | Very High | 43,932.1 | 2024 |
|  | Israel | 0.919 | Very High | 55,690.7 | 2024 |
|  | Qatar | 0.886 | Very High | 126,110.1 | 2024 |
|  | Oman | 0.858 | Very High | 41,664.2 | 2024 |
|  | Kuwait | 0.852 | Very High | 51,636.0 | 2024 |
|  | Egypt | 0.754 | High | 19,094.1 | 2024 |
|  | Lebanon | 0.752 | High | 12,574.8 | 2023 |
|  | South Africa | 0.741 | High | 15,457.5 | 2024 |
|  | Gabon | 0.733 | High | 21,509.50 | 2024 |
|  | Botswana | 0.731 | High | 20,538.1 | 2024 |
|  | Iraq | 0.695 | Medium | 14,464.3 | 2024 |
|  | Namibia | 0.665 | Medium | 11,686.6 | 2024 |
|  | The Democratic Republic of The Congo | 0.649 | Medium | 1,709.7 | 2024 |
|  | Kenya | 0.628 | Medium | 6,619.4 | 2024 |
|  | Ghana | 0.628 | Medium | 8,027.2 | 2024 |
|  | Angola | 0.616 | Medium | 8,348.0 | 2024 |
|  | Cameroon | 0.588 | Medium | 5,591.5 | 2024 |
|  | Uganda | 0.582 | Medium | 3,275.8 | 2024 |
|  | Rwanda | 0.578 | Medium | 3,710.9 | 2024 |
|  | Nigeria | 0.560 | Medium | 6,439.8 | 2024 |
|  | United Republic of Tanzania | 0.555 | Medium | 4,220.8 | 2024 |
|  | Pakistan | 0.544 | Low | 6,287.00 | 2024 |
|  | Malawi | 0.517 | Low | 1,859.4 | 2024 |
|  | Ethiopia | 0.497 | Low | 3,278.5 | 2024 |
|  | Afghanistan | 0.496 | Low | 2,201.7 | 2023 |
|  | Yemen | 0.470 | Low | 3,164.3 | 2013 |
|  | Burkina Faso | 0.459 | Low | 2,896.20 | 2024 |
|  | Mali | 0.419 | Low | 3,308.7 | 2024 |
| **Europe** | Iceland | 0.972 | Very High | 78,258.8 | 2024 |
|  | Switzerland | 0.970 | Very High | 93,818.7 | 2024 |
|  | Norway | 0.970 | Very High | 101,031.6 | 2024 |
|  | Denmark | 0.962 | Very High | 79,514.3 | 2024 |
|  | Gibraltar | 0.961 | Very High | N/A | N/A |
|  | Sweden | 0.959 | Very High | 71,030.5 | 2024 |
|  | Germany | 0.959 | Very High | 72,300.1 | 2024 |
|  | Netherlands | 0.955 | Very High | 84,218.5 | 2024 |
|  | Belgium | 0.951 | Very High | 72,126.0 | 2024 |
|  | Ireland | 0.949 | Very High | 131,175.1 | 2024 |
|  | Finland | 0.948 | Very High | 64,091.2 | 2024 |
|  | United Kingdom | 0.946 | Very High | 60,620.4 | 2024 |
|  | Slovenia | 0.931 | Very High | 56,530.6 | 2024 |
|  | Austria | 0.930 | Very High | 71,617.9 | 2024 |
|  | Malta | 0.924 | Very High | 67,364.2 | 2024 |
|  | Luxembourg | 0.922 | Very High | 150,772.4 | 2024 |
|  | France | 0.920 | Very High | 61,321.7 | 2024 |
|  | Spain | 0.918 | Very High | 56,926.2 | 2024 |
|  | Czech Republic | 0.915 | Very High | 56,805.6 | 2024 |
|  | Italy | 0.915 | Very High | 60,847.0 | 2024 |
|  | Andorra | 0.913 | Very High | 74,939.5 | 2024 |
|  | Cyprus | 0.913 | Very High | 61,239.7 | 2024 |
|  | Greece | 0.908 | Very High | 44,074.3 | 2024 |
|  | Poland | 0.906 | Very High | 50,378.1 | 2024 |
|  | Estonia | 0.905 | Very High | 49,333.8 | 2024 |
|  | Lithuania | 0.895 | Very High | 54,414.0 | 2024 |
|  | Portugal | 0.890 | Very High | 50,616.6 | 2024 |
|  | Croatia | 0.889 | Very High | 48,575.2 | 2024 |
|  | Latvia | 0.889 | Very High | 43,867.0 | 2024 |
|  | Slovakia | 0.880 | Very High | 47,180.8 | 2024 |
|  | Romania | 0.845 | Very High | 48,712.2 | 2024 |
|  | Bulgaria | 0.845 | Very High | 41,086.1 | 2024 |
|  | Georgia | 0.844 | Very High | 28,417.8 | 2024 |
|  | Serbia | 0.833 | Very High | 31,867.2 | 2024 |
|  | Russia | 0.832 | Very High | 47,405.0 | 2024 |
|  | Belarus | 0.824 | Very High | 33,006.4 | 2024 |
|  | Macedonia | 0.815 | Very High | 26,587.0 | 2024 |
|  | Armenia | 0.811 | Very High | 22,823.2 | 2024 |
|  | Albania | 0.810 | Very High | 23,488.0 | 2024 |
|  | Azerbaijan | 0.789 | High | 25,089.0 | 2024 |
|  | Moldova | 0.785 | High | 18,716.5 | 2024 |
|  | Ukraine | 0.779 | High | 18,550.5 | 2024 |
| **Asia Oceania** | Australia | 0.958 | Very High | 71,193.2 | 2024 |
|  | Hong Kong | 0.955 | Very High | 75,215.7 | 2024 |
|  | Singapore | 0.946 | Very High | 150,689.3 | 2024 |
|  | New Zealand | 0.938 | Very High | 55,093.5 | 2024 |
|  | Korea | 0.937 | Very High | 52,204.0 | 2023 |
|  | Japan | 0.925 | Very High | 51,685.0 | 2024 |
|  | Taiwan | 0.925 | Very High | N/A | N/A |
|  | Guam | 0.901* | Very High | N/A | N/A |
|  | Brunei Darussalam | 0.837 | Very High | 90,007.1 | 2024 |
|  | Kazakhstan | 0.837 | Very High | 40,813.0 | 2024 |
|  | Malaysia | 0.819 | Very High | 38,728.9 | 2024 |
|  | Thailand | 0.798 | High | 24,708.2 | 2024 |
|  | China | 0.797 | High | 27,104.9 | 2024 |
|  | Sri Lanka | 0.776 | High | 15,632.6 | 2024 |
|  | Vietnam | 0.766 | High | 16,385.5 | 2024 |
|  | Mongolia | 0.747 | High | 19,097.7 | 2024 |
|  | Uzbekistan | 0.740 | High | 11,878.6 | 2024 |
|  | Fiji | 0.731 | High | 16,032.3 | 2024 |
|  | Indonesia | 0.728 | High | 16,448.3 | 2024 |
|  | Philippines | 0.720 | High | 11,794.1 | 2024 |
|  | Kyrgyzstan | 0.720 | High | 8,009.4 | 2024 |
|  | American Samoa | 0.708 | High | N/A | N/A |
|  | India | 0.685 | Medium | 11,158.9 | 2024 |
|  | Bangladesh | 0.685 | Medium | 9,646.8 | 2024 |
|  | Nepal | 0.622 | Medium | 5,736.6 | 2024 |
|  | Myanmar | 0.609 | Medium | 5,997.5 | 2024 |
|  | Papua New Guinea | 0.576 | Medium | 4,888.6 | 2024 |
|  | United States Minor Outlying Islands | N/A | N/A | N/A | N/A |
| **North America** | Canada | 0.939 | Very High | 65,463.1 | 2024 |
|  | United States | 0.938 | Very High | 85,809.9 | 2024 |
| **Latin America**  **254(16.7%)** | Aruba | 0.879* | Very High | 44,967.3 | 2023 |
|  | Chile | 0.878 | Very High | 34,637.1 | 2024 |
|  | Argentina | 0.865 | Very High | 30,175.5 | 2024 |
|  | Uruguay | 0.862 | Very High | 36,417.9 | 2024 |
|  | Antigua and Barbuda | 0.851 | Very High | 33,602.4 | 2024 |
|  | Puerto Rico | 0.845 | Very High | 50,155.5 | 2024 |
|  | Panama | 0.839 | Very High | 41,404.8 | 2024 |
|  | Costa Rica | 0.833 | Very High | 30,062.8 | 2024 |
|  | Peru | 0.794 | High | 17,802.4 | 2024 |
|  | Mexico | 0.789 | High | 25,688.1 | 2024 |
|  | Colombia | 0.788 | High | 21,494.6 | 2024 |
|  | Brazil | 0.786 | High | 22,333.4 | 2024 |
|  | Ecuador | 0.777 | High | 15,840.3 | 2024 |
|  | Guyana | 0.776 | High | 79,905.7 | 2024 |
|  | Dominican Republic | 0.776 | High | 27,541.2 | 2024 |
|  | Cuba | 0.762 | High | N/A | N/A |
|  | Paraguay | 0.756 | High | 18,523.7 | 2024 |
|  | Bolivia | 0.733 | High | 11,189.8 | 2024 |
|  | Belize | 0.721 | High | 15,092.8 | 2024 |
|  | Venezuela | 0.709 | High | 17,349.2 | 2011 |
|  | Guatemala | 0.662 | Medium | 14,368.7 | 2024 |
|  | Monserrat | N/A | N/A | N/A | N/A |
|  | Guadeloupe | N/A | N/A | N/A | N/A |
| **Other** | Antarctica | N/A | N/A | N/A | N/A |
| **References** | [**https://worldpopulationreview.com/country-rankings/hdi-by-country#title**](https://worldpopulationreview.com/country-rankings/hdi-by-country#title) Accessed August 14, 2025  [**https://data.worldbank.org/indicator/NY.GDP.PCAP.PP.CD?name_desc=false**](https://data.worldbank.org/indicator/NY.GDP.PCAP.PP.CD?name_desc=false) Accessed August 14, 2025 | | | | |

| **Supplementary Table 6: Participants’ nutritional risk status based on the FIGO Nutrition Checklist answers according to the FIGO Five regions. N=1505** | | | | | | | | |
| --- | --- | --- | --- | --- | --- | --- | --- | --- |
|  | **Total** | **Africa and Eastern Mediterranean** | **Asia Oceania** | **Europe** | **Latin America** | **North America** | **Other** | **P-value** |
|  | N = 1505 | N = 88 | N = 401 | N = 673 | N = 253 | N = 89 | N = 1 |  |
| **At nutritional risk, n (%)** | 1423 (94.6%) | 81 (92%) | 378 (94.3%) | 644 (95.7%) | 236 (93.3%) | 83 (93.3%) | 1 (100%) | 0.553 |
| **Not at nutritional risk, n (%)** | 82 (5.4%) | 7 (8%) | 23 (5.7%) | 29 (4.3%) | 17 (6.7%) | 6 (6.7%) | 0 |  |
| *“At nutritional risk” is indicative of having answered “No” to at least one question on the FIGO Nutrition Checklist. “Not at nutritional risk” indicates all “yes” answers to the FIGO Nutrition Checklist.* | | | | | | | | |

| **Supplementary Table 7: Achieving dietary standards in the FIGO Five Regions. N = 1505** | | | | | | | |
| --- | --- | --- | --- | --- | --- | --- | --- |
|  | **Africa and Eastern Mediterranean** | **Asia Oceania** | **Europe** | **Latin America** | **North America** | **Other** | **P-value** |
|  | N = 88 | N = 401 | N = 673 | N = 253 | N = 89 | N = 1 |  |
| ***Do you eat three or more servings of fruit or vegetables (dried, juice, frozen, tinned or fresh) each day?*** | | | | | | | |
| ‘Yes’, n (%) | 52 (59%) | 214 (53%) | **463 (68.7%)** | 151 (59.6%) | 57 (64%) | 0 (0%) | **<0.001** |
| ***Do you eat at least one serving of dairy products (milk/milky drinks, yoghurt, cheese) each day?*** | | | | | | | |
| ‘Yes’, n (%) | 62 (70%) | 249 (62%) | 496 (73.7%) | **205 (81%)** | 64 (71.9%) | 1 (100%) | **<0.001** |
| ***Do you eat at least one serving of wholegrain or "brown" versions of foods such as bread, rice, pasta, crackers, cereals, oats, millet or wheat each day?*** | | | | | | | |
| ‘Yes’, n (%) | 69 (78.4%) | 301 (75%) | **554 (82%)** | 198 (78%) | 68 (77.5%) | 0 (0%) | 0.029 |
| ***Do you eat at least two or three servings of meat, poultry (chicken or turkey) or eggs each week?*** | | | | | | | |
| ‘Yes’, n (%) | 76 (86%) | 294 (73%) | 576 (85.5%) | **229 (90.5%)** | 73 (82%) | 0 (0%) | **<0.001** |
| ***Do you eat at least two or three servings of pulses, beans, peas, lentils, nuts or seeds each week?*** | | | | | | | |
| ‘Yes’, n (%) | 58 (65.9%) | 237 (59%) | 281 (41.7%) | 184 (72.7%) | **73 (82%)** | 1 (100%) | **<0.001** |
| ***Do you eat at least one serving of fish each week?*** | | | | | | | |
| ‘Yes’, n (%) | 38 (43%) | 210 (52%) | 387 (57.5%) | 132 (52%) | **53 (59.5%)** | 0 (0%) | 0.065 |
| ***Do you limit eating packaged snacks, cakes, pastries, or sugar-sweetened drinks to less than five times a week?*** | | | | | | | |
| ‘Yes’, n (%) | **64 (72.7%)** | 254 (63%) | 417 (61.9%) | 173 (68%) | 61 (68.5%) | 0 (0%) | 0.116 |
| ***Do you take folic acid?*** | | | | | | | |
| ‘Yes’, n (%) | 39 (44%) | 148 (36.9%) | 313 (46.5%) | 97 (38%) | **43 (48%)** | 0 (0%) | 0.028 |
| ***Do you get regular exposure to the sun on your face, arms and hands for at least 10-15 minutes a day?*** | | | | | | | |
| ‘Yes’, n (%) | 57 (64.7%) | 233 (58%) | 295 (43.8%) | 135 (53%) | **61 (68.5%)** | 0 (0%) | **<0.001** |
| *Data represented as categorical variables reported as n, (%) based on FIGO region. Values determined using statistical significance p-value <0.05.* | | | | | | | |

| **Supplementary Table 8: Proportion of respondents to the FIGO Nutrition Checklist who reported following a special diet. N = 342** | | | | | | | |
| --- | --- | --- | --- | --- | --- | --- | --- |
|  | **Total** | **Africa and Eastern Mediterranean** | **Asia Oceania** | **Europe** | **Latin America** | **North America** | **Other** |
| **Pescatarian, n (%)** | 74 (21%) | 4(4.5%) | 23(5.7%) | 37(5.5%) | 3(1.2%) | 7(7.9%) | 0 |
| **Flexitarian, n (%)** | 82 (23.9%) | 5(5.7%) | 31(7.7%) | 26(3.9%) | 12(4.7%) | 8(9%) | 0 |
| **Vegetarian, n (%)** | 88 (25.7%) | 5(5.7%) | 48(11.9%) | 21(3.1%) | 11(4.4%) | 3(3.3%) | 0 |
| **Vegan, n (%)** | 50 (14.6%) | 0 | 12(3%) | 26(3.9%) | 6(2.4%) | 6(6.7%) | 0 |
| **Other, n (%)*** | 48 (14%) |  |  |  |  |  |  |
| **Other represents special diets not included in analysis.* | | | | | | | |
